# Protein quality and quantity control at the yeast ER

**DOI:** 10.18632/oncotarget.4855

**Published:** 2015-07-14

**Authors:** Stefan G. Kreft

**Affiliations:** Department of Biology, University of Konstanz, Konstanz, Germany

Protein degradation constitutes a central pillar in proteome maintenance. In eukaryotes, specific protein turnover is predominantly achieved by the ubiquitin-proteasome system (UPS). Therein, substrate proteins are first covalently decorated with the small protein ubiquitin. Sequential addition of ubiquitin monomers results in a polyubiquitin chain on a substrate, the signal for proteasomal degradation. Attachment of ubiquitin(s) proceeds via an enzymatic cascade involving ubiquitin-activating enzyme(s) (E1), ubiquitin-conjugating enzymes (E2), and ubiquitin-protein ligases (E3). Specificity relies chiefly on the E3 ligases. Most integral membrane and secretory proteins are synthesized at the ER where they are surveilled by sophisticated ER quality control machinery. Misfolded or otherwise aberrant membrane or secretory proteins are terminally removed by UPS-dependent degradation in a process termed ER-Associated Degradation (ERAD). ERAD not only operates in protein quality control but also plays a central role in protein quantity control. In the latter case, ERAD maintains the appropriate abundance of specific proteins. Membrane-embedded E3 ligase complexes coordinate ERAD substrate recognition, retrotranslocation to the cytosolic face of the ER membrane, and ubiquitylation (which occurs in the cytosol). Depending on the localization of the degradation signal (degron), ERAD substrates have historically been classified into three main categories: ERAD-L (lumen), ERAD-M (membrane), and ERAD-C (cytosol).

In yeast, the two major ERAD E3 complexes are the Hrd1/Der3 complex and the Doa10 complex; homologous complexes exist in metazoans. For almost a decade, the prevailing notion (based on a limited number of substrates) has been that ERAD-L and ERAD-M substrates are handled exclusively by Hrd1, whereas ERAD-C substrates are the domain of Doa10.

Our recent studies have revealed that, contrary to the prevailing view, Doa10 can also function in ERAD-M [1]. The intramembrane degron recognized by Doa10 resides in the tail-anchor (TA) region of the integral membrane protein Sbh2, the β-subunit of the heterotrimeric Ssh1 translocon complex in the yeast ER membrane. Previous work established that Sbh2 levels are decreased in cells lacking its binding partner Ssh1 [2]. Consistent with this, we identified unassembled Sbh2 as a bona fide Doa10 substrate. We further demonstrated that Doa10 recognizes Sbh2 following membrane insertion. This was important to demonstrate, as some membrane proteins are recognized prior to membrane insertion via a preemptive Doa10-dependent ERAD-C-related mechanism [3]. After the identification of Sbh2 as a Doa10 substrate, the precise nature and position of the degron within unassembled Sbh2 was examined. For this endeavor, chimeric proteins consisting of portions of Sbh2 and its metabolically stable homolog Sbh1 proved immensely valuable. The use of Sbh1-Sbh2 chimeric proteins (as well as truncated variants of Sbh2) allowed us to map the degradation signal to the TA region of Sbh2 (comprising the transmembrane (TM) helix and 6-residue ER luminal polypeptide segment). These experiments were consistent with an intramembrane degron within unassembled Shb2 that targets the protein for Doa10-dependent degradation. Confirmation of the presence of an intramembrane degron emerged from experiments with Sbh2 bearing single point mutations in the TM region. The amino acid sequences of the TMs of Sbh1 and Sbh2 are highly similar. A striking difference between the two is a single residue in the TM of Sbh2 (Ser68); an alanine residue is found at the corresponding position in Sbh1. Mutation of Sbh2 Ser68 to alanine partially stabilized the Sbh2(S68A) mutant. This serine-to-alanine exchange did not detectably increase the affinity for Sbh1's binding partner Sec61 (one possible explanation for stabilization). Thus, the partial stabilization observed for Sbh2(S68A) demonstrated that Ser68 is a crucial part of the degron. Together, these experiments establish Sbh2 as the first Doa10 substrate that possesses an intramembrane degron. Moreover, they demonstrate that Doa10 has an intrinsic capacity to recognize specific intramembrane degrons. Future research will show whether additional ERAD-M degrons are recognized by Doa10.

An important remaining question is how Doa10 recognizes the Sbh2 intramembrane degron. Hrd1-dependent ERAD-M degrons often display hydrophilic residues within their TM region, and these are believed to be directly recognized by hydrophilic TM residues of Hrd1 [4]. Several hydrophilic residues are also present within the 14 TM helices of Doa10 [5], consistent with direct recognition by Doa10 of the Sbh2 intramembrane degron. Notably, a second TA protein, the E2 enzyme Ubc6 (one of the two cognate E2s that function with Doa10) is also a Doa10 substrate. However, while Ubc6 TA sequence is necessary for interaction with Doa10, it is not sufficient to promote Doa10-dependent degradation (*i.e.* it does not represent a degron per se) [6]. The different behavior of Doa10 toward the Sbh2 and Ubc6 TA sequences highlights the multifaceted interactions of Doa10 with TA sequences and intramembrane degrons.

Targeting degradative pathways represents an attractive strategy for treating a range of human diseases [7]. A deeper mechanistic understanding of substrate recognition by ERAD E3 complexes may thus pave the way for novel approaches to manipulating degradation of specific medically relevant substrates.
